# Annotation and visualization of parasite, fungi and arthropod genomes with Companion

**DOI:** 10.1093/nar/gkae378

**Published:** 2024-05-16

**Authors:** William Haese-Hill, Kathryn Crouch, Thomas D Otto

**Affiliations:** School of Infection & Immunity, University of Glasgow, UK; School of Infection & Immunity, University of Glasgow, UK; School of Infection & Immunity, University of Glasgow, UK; LPHI, CNRS, INSERM, Université de Montpellier, France

## Abstract

As sequencing genomes has become increasingly popular, the need for annotation of the resulting assemblies is growing. Structural and functional annotation is still challenging as it includes finding the correct gene sequences, annotating other elements such as RNA and being able to submit those data to databases to share it with the community. Compared to *de novo* assembly where contiguous chromosomes are a sign of high quality, it is difficult to visualize and assess the quality of annotation. We developed the Companion web server to allow non-experts to annotate their genome using a reference-based method, enabling them to assess the output before submitting to public databases. In this update paper, we describe how we have included novel methods for gene finding and made the Companion server more efficient for annotation of genomes of up to 1 Gb in size. The reference set was increased to include genomes of interest for human and animal health from the fungi and arthropod kingdoms. We show that Companion outperforms existing comparable tools where closely related references are available.

## Graphical Abstract

**Graphical Abstract:**
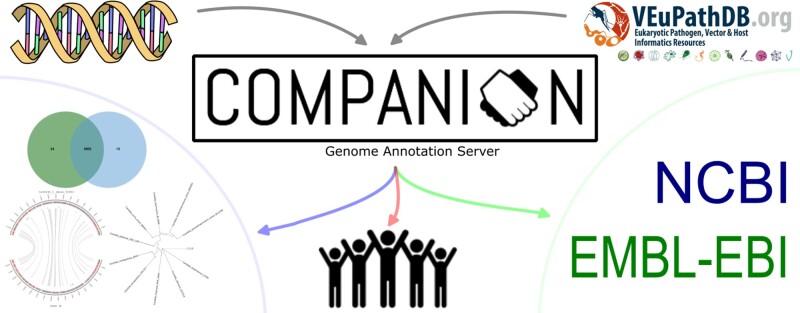

## Introduction

Over the last 15 years, the maturation of long-read sequencing technologies, the decreasing cost of sequencing, together with the development of simple and efficient software for *de novo* assembly (1,2) has enabled the community to produce contiguous assemblies for species with repetitive or low complexity genomes that were fragmented and incomplete with short-read technologies. These advances inspired ambitious proposals such as the Earth BioGenome Project (EBGP) to sequence ∼1.5 million of the current estimated 10–15 million Eukaryotic species (3).

Although it has become increasingly easy to assemble genomes, the annotation of assemblies remains a difficult problem and is often neglected. For example in Brůna et al. (4), as the best performing tool, BRAKER2 ranges for exon-level sensitivity and specificity in three applications between 77–85% and 85–91%, respectively. Although there was an increase of sensitivity for *ab initio* gene finding due to long read RNA-Seq, which enables discovery of alternative splicing events, it is still an open question to find the correct start codon. Further, there are limitations to determining gene models in regions without strong RNA-Seq evidence, resulting in the overall accuracy of gene models generated with long RNA-Seq reads being still under 90% (5). Further, in a full annotation process, many different tools are involved; whether gene finding, functional annotation, or ncRNA detection. There are additional challenges in submitting the annotation to the International Nucleotide Sequence Database Collaboration (INSDC) (GenBank, EMBL-EBI and DDBJ) due to an overly stringent pipeline for uploading the data. This results in poor public availability of genome annotations and data that does not uphold Findable, Accessible, Interoperable and Reusable (FAIR) principles (6).

To overcome the above issues, several tools have been developed to perform automated genome annotation. However, some tools, such as CAT (7), FunAnnotate (Palmer and Stajich, 2023) and the NCBI Eukaryotic Annotation Pipeline (8), lack web-based or graphical interfaces, raising the barrier to tool use. The NCBI pipeline requires a user to send an email requesting annotation, centralising the annotation process rather than democratising it, and when we requested the annotation of a parasite genome, this was not possible. Easier to use are webservers, like GenSAS (9), MEGANTE (10) and WebAUGUSTUS (11). However, MEGANTE is limited by a maximum upload file size of 10MB and lacks appropriate training models for species like Fungi. WebAUGUSTUS offers a greater choice of Fungi references but minimal Protozoan parasites and has a limited scope. GenSAS works without restrictions for most components of its pipeline (some require additional evidence, e.g. RNA-Seq data for its implementation of BRAKER) and contains a wide range of pretrained models for structural annotation. However, it lacks some parasite reference sets, does not have a graphical output, and relies on expert knowledge to set up.

In 2016, we developed ‘Companion’ version 1 (12). The web server provides an easy-to-use pipeline for reference-based annotation with pre-compiled reference genomes derived from VEuPathDB (13). Companion uses ABACAS (14) to scaffold contigs against a reference, and can then transfer the high quality annotations from the reference to the new assembly, using RATT (15). Transfer of annotations is less error prone than *ab initio* gene prediction, if the two genomes are similar to each other (>95% nucleotide identity) (15). However, where annotations cannot be transferred, *ab initio* gene prediction is performed with AUGUSTUS (16). Companion supports the use of RNA-Seq data to improve annotations. Output can be downloaded in a selection of formats that have been designed to facilitate upload to the European Nucleotide Archive (ENA) of EMBL-EBI. A particular strength of Companion is the visual output which includes basic statistics, a phylogenetic tree generated against the reference set, analysis of orthology with OrthoMCL (17) to assess the quality of the annotation, and synteny maps to assess the completeness of the assembly.

Despite its small parasite user community, Companion version 1 was heavily used and with the increasing number of assemblies generated in the communities of vectors and arthropods, we decided to extend the range of references to those. Early testing with these additional, often much larger, reference genomes revealed numerous pipeline components that were unable to scale efficiently, and so needed updating to ensure a satisfactory outcome.

Here, we describe the implementation improvements to not only enable the annotation of larger genomes (included in the set of 438 reference genomes available on the web server, Supplementary Table S1), but also to improve robustness and ensure faster runtimes. Finally, we show that Companion generally outperforms another web-based annotation software tool, GenSAS, for reference-based annotation of fungal, protozoan parasite, and vector genomes, and should be considered as a tool for the community to annotate such species going forward.

## Materials and methods

### Updated workflow

Companion version 2 is implemented as a Nextflow DSL1 pipeline and the source code is available at https://github.com/sii-companion/companion. GitHub Actions is used for automated testing and tag/release generation, where releases follow the standard semantic versioning format. For running locally, a docker container is hosted at https://hub.docker.com/repository/docker/uofgiii/companion/ with builds synced to each code release. Instructions are available on the GitHub Wiki for building a bespoke reference dataset when running a container locally. This ensures that users are not restricted to reference genomes hosted on the web server, which is currently restricted to genomes available at VEuPathDB.

**Figure 1.**
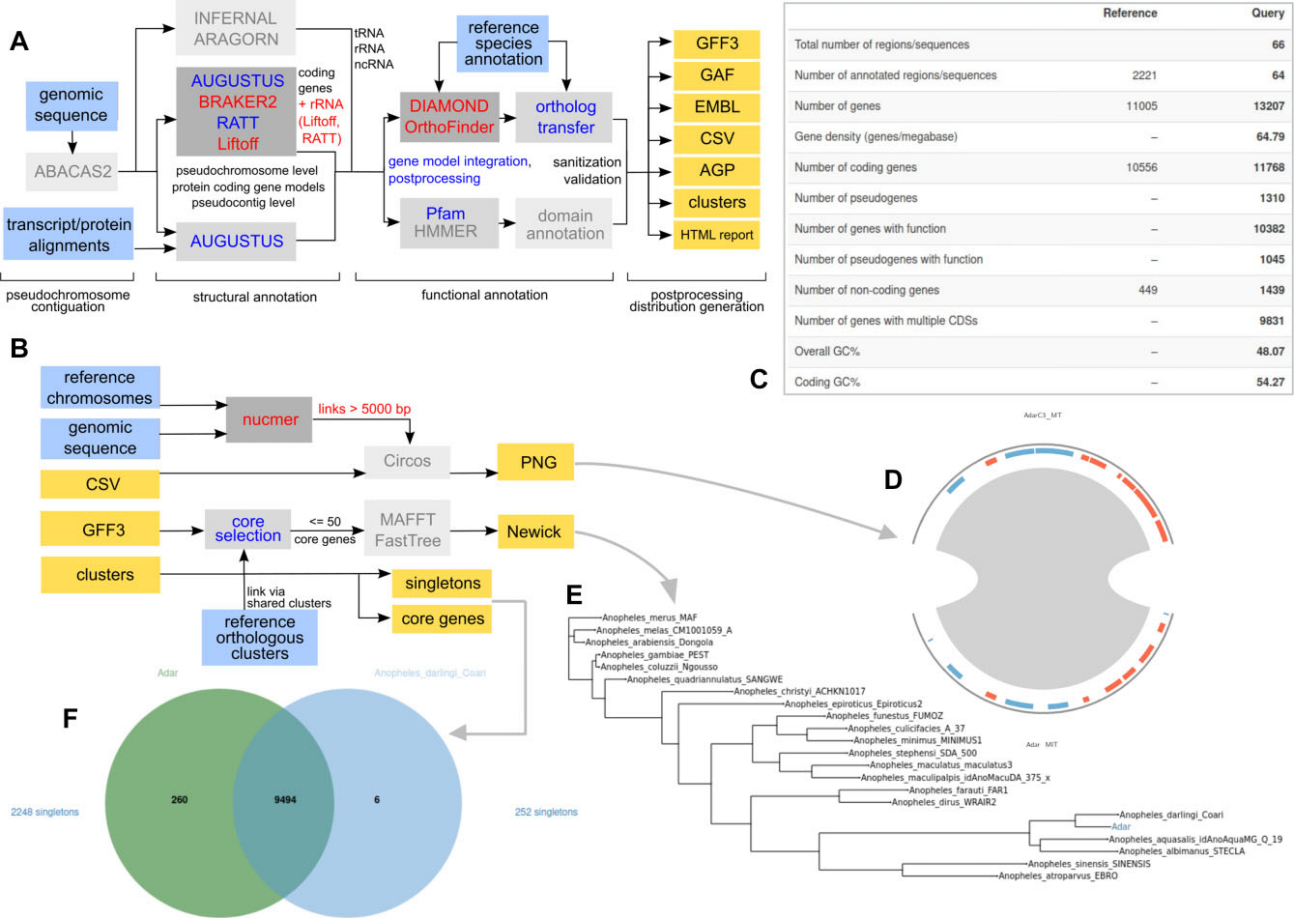
Companion workflows as first shown in Figure 1 from the original paper (12), where additional components are highlighted with red text and updated components with blue text. All other components (with grey text and lighter-shaded boxes) are largely unchanged since Companion version 1. (**A**) Genome annotation workflow. (**B**) Downstream analysis and visualization workflow. Input files are represented as blue boxes, output files as yellow boxes. All output files are used to construct the result set presented in the web interface, example given of *Anopheles darlingi* assembly target: (**C**) annotation summary statistics between reference and target; (**D**) target-reference synteny for mitochondrial chromosome; (**E**) phylogenetic tree placing newly annotated species (here ‘Adar’) in the context of the *Anopheles* genus; (**F**) interactive Venn diagram summarizing core and species-specific clusters. Summaries of all workflow tools are available in a glossary in the Supplementary Data, and additional visualizations for an example *Candida dubliniensis* job can be seen in Supplementary Figure S6.

Compared to the first version v1.0.2, the current version v2.2.4 has several new features implemented (see Figure 1 – red text) and others improved (blue text). These changes allow annotation of genomes up to 3 Gb, although we observed optimal performance in genomes up to 1 Gb.

As justified in the Supplementary Data, BRAKER2 v2.1.6 (4) was included in the pipeline and can be used as a default option (over AUGUSTUS). Due to the challenges of incorporating a multi-faceted pipeline like BRAKER2, Companion has additional infrastructure to ensure AUGUSTUS is utilized as a backup in the event of process failure. BRAKER2 is called in protein data mode (see workflow description https://github.com/Gaius-Augustus/BRAKER#braker-with-protein-data), using annotated protein sequences gathered from the reference species family.

To ensure more efficient usage of memory and faster runtimes, we updated RATT, OrthoMCL, BLASTP and BLASTN with Liftoff v1.6.3 (18), OrthoFinder v2.5.4 (19), DIAMOND (20) and nucmer from MUMmer4.x (21) v4.0.0rc1 (latest), respectively (see Figure 1). While RATT and Liftoff produce comparable results for most genomes with high similarity to their reference (Supplementary Table S3), only Liftoff could function with the larger vector genomes introduced to the web server.

For further information and justification for pipeline component upgrades, see Supplementary Data.

### Web server

The web application is implemented in Ruby on Rails with a MySQL database, hosted on a server with 32 CPU cores, 64 GB RAM, as well as 3 TB storage. It can be accessed from https://companion.ac.uk/. Three jobs can be run concurrently. Active development is carried out on a development server with matching resources, where full testing of updated features can be carried out without impacting production queues. There is also a build server for integration testing of new Companion release candidates, which has 8 CPU cores, 8 GB RAM and 200 GB storage, with only single job concurrency.

For further information about the web server enhancements, see Supplementary Data.

### Testing against alternative web server

To test comparisons with another tool, we used the Companion web server running v2.2.0 and GenSAS v6.0. The input nucleotide sequence fasta file used for each test was identical for both tools. Unless otherwise stated, it can be assumed that default settings were used for each test in the case of Companion, while components were selected in GenSAS to ensure the best possible outcome of results with the limited input data available.

For GenSAS, we created an account and selected the closest AUGUSTUS reference available for structural annotation. While GenSAS also offers BRAKER for structural annotation (which, as the Companion default, would have been a preferable option for comparison), unlike Companion its implementation requires additional RNA-Seq evidence, which was not available for the tests. During a naïve first attempt, we selected additional options in preceding tabs (including repeat masking and alignment) on the understanding that these should be expected as part of the pipeline. However, subsequent testing revealed that, while steps such as repeat masking can make a dramatic difference in annotation quality for many eukaryotic species (22), they dramatically increased runtime with negligible (if any) improvement to accuracy in our tests, and so were omitted from the final results.

References for Companion were selected based on their close similarity to the target strain. The target and reference species, along with their VEuPathDB release numbers, are shown in Supplementary Table S2. Canonical annotations in GFF format were also available to use from each of these releases.

Structural comparison of outputs with the canonical annotations were performed using GffCompare (23) where such a reference was available, utilizing standard metrics such as precision and sensitivity, similar to those used in Holt and Yandell (24) (where the term ‘accuracy’ is used to define the mean of precision and sensitivity values). We focus on the following metrics, as defined explicitly in Pertea and Pertea (23):

- Nucleotide accuracy, which refers to the proportion of overlapping bases of a gene between the two comparators.
- Exon accuracy, as before but determined by overlapping and matching exon boundaries between the two comparators.
- Matching loci, where all the coordinates of a gene are identical between the two comparators (including all its exon) - a perfect match.
- Total genes (including pseudogenes), which can be used to determine if genes are being overpredicted by either tool.

While both pipelines also provide functional annotation outputs, an objective metric of their quality versus the canonical annotation could not be identified.

## Results

### Motivation for Companion version 2

With Companion version 1, and before the improvements to the web server, we had ∼100 unique users annotating ∼1000 genomes annually. Since version 2 these annual figures have doubled (annotating ∼2000 genomes). It can be observed that each user might annotate several genomes or rerun annotation jobs with alternative settings.

### Performance versus GenSAS

To assess the claim that the latest Companion release provides the most user-friendly and accurate annotation platform for a user with minimal additional evidence and a target species closely related to the reference species included in Companion, we performed three tests ranging from different species complexes and compared them with the web application GenSAS.

First, we tested how easy it was to use each server. It was noted that Companion needed just 8 clicks, while GenSAS needed 41 (Supplementary Figure S1) to submit a basic job. Further, in Companion all settings are displayed on a single page (Supplementary Figure S2), rather than requiring the user to click through multiple tabs as in GenSAS, making Companion (in our view) more user-friendly for those unfamiliar with annotation pipelines.

We ran a series of jobs with matching input genomes on both Companion and GenSAS, aiming to compare annotation outputs to a canonical annotation obtained from VEuPathDB. The assumption in each case was that a naïve user might only have the assembly sequence available with no additional evidence and would tend to choose default settings; a quality annotation should be expected regardless. To converge on a good breadth and high-quality reference dataset, we chose the reference genomes from VEuPathDB. The remit of VEuPathDB is to facilitate as many researchers as possible from the communities of parasites, fungi and arthropods to cover their bioinformatics needs.

*Parasite: Plasmodium*. As the original release of Companion specialized in annotation of Apicomplexa, including *Plasmodium*, it seemed worthwhile to consider a *Plasmodium* test with a closely related target and reference strain, to demonstrate both the continued supremacy of Companion for such genomes but also how much it has improved.

*Plasmodium falciparum* Dd2 was chosen as a target due to having a manually curated canonical annotation available (25), and as a species match to the reference *Plasmodium falciparum* 3D7 (26): a consistently popular reference selection amongst Companion users (accounting for > 25% of all job submissions over the previous six months). This represents a comparison of very similar genomes with divergent subtelomeric regions (5%). Within GenSAS, BRAKER requires uploading additional RNA-Seq data in BAM format, while their implementation of AUGUSTUS doesn’t contain a *Plasmodium* training set, so we were forced to use GeneMarkES for (*ab initio*) structural annotation – this was the only other structural tool on offer that didn’t require additional evidence.

Overall, Companion outperforms GenSAS where closely related references are available, see Table 1. The advantages of a reference-based approach for transferring gene models are evident, especially when considering the ∼30% difference in matching loci. Additionally, GenSAS fails to predict ∼8% of the total genes that are present in the canonical annotation; Supplementary Figure S3 shows that most gene orthogroups are found by Companion, with many missed by GenSAS. The lower number of genes and matching loci, despite high nucleotide accuracy, in GenSAS can be partially explained by its incorrect merging of several transcripts as a single gene, see Supplementary Figure S4. In the same figure it can also be observed that GenSAS fails to call smaller exons. Furthermore, Companion version 2 has seen a noticeable improvement in its detection of apicoplast and mitochondrial genes over both version 1 and GenSAS (Supplementary Figure S5).

**Table 1.** Standard metrics to compare performance of GenSAS and Companion against canonical annotations, for various organisms

Organism	Metric	GenSAS	Companion
*Plasmodium falciparum* ^‡^	Nucleotide accuracy (%)	98.65	99.35
(5461 total genes)	Exon accuracy (%)	79.35	95.05
	Matching loci (%)	66.64	96.22
	Total genes predicted	5034	5800
	Runtime (h)	6/1*	2.5
*Candida dubliniensis*	Nucleotide accuracy (%)	98.55	98.35
(6095 total genes)	Exon accuracy (%)	85.95	89.50
	Matching loci (%)	90.85	95.54
	Total genes predicted	5822	6206
	Runtime (h)	17.5/1*	1
*Anopheles darlingi* ^†^	Nucleotide accuracy (%)	87.1	86.15
(12 393 total genes)	Exon accuracy (%)	66.75	74
	Matching loci (%)	48.27	65.05
	Total genes predicted	14429	16500
	Runtime (h)	13.5/3*	9

^†^Comparison of CDS features only due to presence of UTR features in canonical annotation.

^‡^Removed UTR features from Companion output due to absence in canonical annotation.

*Times given for full pipeline and minimal pipeline jobs (see Supplementary Data). Referenced statistics are from the latter.

Accuracy is mean of sensitivity and precision values. Note significant improvement in exon accuracy and matched loci for Companion in all cases, as well as greater number of total genes predicted.

While our focus is on genome annotation with minimal additional input data, both tools can include additional RNA-Seq evidence (Supplementary Table S4). It can be observed that, as expected, results with RNA-Seq evidence are generally improved (significantly for GenSAS). However, Companion still outperforms GenSAS over virtually all metrics, whether RNA-Seq data has been included or not.

*Fungi: Candida*. Companion's reference database has been expanded to include all available species from FungiDB (Supplementary Table S1), which includes fungal pathogens of humans, animals and plants, as well as model organisms. We decided to use *Candida dubliniensis* CD36 (27) as a target species against the reference *Candida albicans* SC5314 (28) as this would allow testing performance where there is slightly more phylogenetic distance than in the previous test (albeit the same genus). Once more, there was a well-annotated canonical annotation hosted on FungiDB (part of VEuPathDB).

This time, we noted that the AUGUSTUS training set offered by GenSAS contained a *Candida albicans* reference, so this was selected for structural annotation. The results of an almost identical run (but with RATT instead of Liftoff and using Companion v2.0.10) can also be seen with all outputs and visualizations at https://companion.gla.ac.uk/examples. Some of these outputs are depicted in Supplementary Figure S6, demonstrating how closely related the genomes are and how well the interface helps the user to understand the differences between the reference and query.

As GenSAS had a pre-trained model, the difference between the two runs was not as striking, e.g. Companion is just 5% better in matching loci (Table 1). Interestingly, for GenSAS the number of genes predicted was low (∼300 less than the reference), however the nucleotide accuracy is marginally higher than in Companion. This can be explained by GenSAS merging genes. Once again, consistent improvement in the standardized metrics can be observed for Companion.

*Vector: Anopheles*. The final test was with the *Anopheles darlingi* vector (Table 1 and Supplementary Data). It should be noted, although the two assemblies are relatively closely related (median identity of 1–1 orthologous proteins is 99.6%), the reference genome is in 2221 supercontigs and has a size of 133 Mb, while the new improved query has a size of 177 Mb in 66 supercontigs. In terms of comparison between Companion and GenSAS, the results follow a similar narrative as the Fungi test: Companion achieves higher exon accuracy and matching loci, GenSAS runs faster with minimal settings and has higher nucleotide accuracy. However, both methods overpredicted the number of genes and we observed an overall drop in performance compared to the two previous tests. This could due to the fact that the VEuPathDB reference used by Companion (Supplementary Table S2) is not complete, highlighting the limitation of annotation if a well-annotated reference genome is not available. As a new metric we considered functional annotation of Pfam domains (Supplementary Figure S7), where we could show that the genes predicted by Companion have a >2% higher coverage.

In conclusion, where good reference genomes are available (*Plasmodium* and *Fungi*) Companion works well, especially when reference and query are closely related (the median identity of 1–1 orthologous proteins for the *Plasmodium* reference and query is 99.8%, while in the Fungi it is 90.6%). For the vector, Companion still performs comparably to other pipelines. However, although there is high similarity in sequence, the current reference of *Anopheles darlingi* might not be of enough high quality (fragmented, no manual curation) for reference-based annotation.

## Discussion

Projects associated with the Earth BioGenome Project (EBGP) promise to build the genomes of 4 million species – but how will they be annotated? The Darwin Tree of Life have their own bespoke pipeline aiming to generate high quality genome assemblies (telomer to telomer) and build the annotation with RNA-Seq evidence in Ensembl (29). However, other projects such as the European Reference Genome Atlas (ERGA) (30) are more community driven with >700 groups contributing *de novo* assemblies (and not necessarily with RNA-Seq evidence). It would be inefficient for each group to generate their own annotation pipeline. Further, we expect that high quality reference genomes (contiguous and RNA-Seq evidence even manual annotation) of projects like the Darwin Tree of Life can be used in Companion for reference-based annotation. It can be observed that this is an ongoing process, as the current vector reference genome for *Anopheles darlingi* in VEuPathDB (used in our comparison) is of lower quality than the newly generated genome assembly from the Darwin Tree of Life. This suggests that with an increase in high quality genomes, Companion will become more useful for annotating closely related species.

In terms of sustainability, we argue that it is far more economical to have a tested service rather than building and testing new pipelines on an ad-hoc basis. Although it is straightforward to upload the sequence of the assembly to ENA, a mechanism for easily uploading genome annotations into the main databases is lacking. EMBL-EBI is trying to overcome these issues, as the GFF format is an open file format, though including it into databases is a time-consuming and manual process. The consequences are that genome sequences without annotation are submitted to the databases, with the risk that the annotation might not be accessible under FAIR principles.

With Companion we present a solution to this dilemma; a service easy to run, freely available, including visualisation options and producing high quality annotations. Although the main strength of Companion comes from the reference-based annotation (Table 1), with the introduction of BRAKER2, we have implemented a tool that can efficiently self-train with protein evidence. However, many groups in the community are interested in the study of diversity of species complexes, understanding the evolution of species, and exploration of specific traits such as antimicrobial resistance. Looking at the assembly statistics, many isolates from a well-annotated reference e.g. *Plasmodium*/*Trypanosome* were annotated in the last years with Companion. Indeed, in the last 6 months alone there have been 275 successful Companion web server jobs with a *Plasmodium* reference. This reflects the goals of researchers to frequently sequence multiple isolates from a single species or from related species, and here the transfer of annotation from a reference is of huge advantage—a unique hallmark of Companion. Although Companion has a predefined reference set, 300 unique users have downloaded the stand-alone Docker container since the release of version 2, which allows the use of bespoke reference datasets not contained within VEuPathDB.

It should be noted that annotation is an open problem. For example, even using stand-alone BRAKER2 or MAKER2 with protein and RNA-Seq evidence, the exon accuracy is generally under 80% for species such as vectors. This explains why for key species such as mouse, human, and *Plasmodium*, there are efforts of manual curation. In the absence of those, Companion generates good first pass annotation as shown for the vector test (Table 1).

In conclusion, we present a reference-based annotation web server for the community to overcome the on-going difficulties of genome annotation. In recent years, we have tripled our usage (Supplementary Data) and expect with the increase of phyla to reach even more communities.

## Supplementary Material

gkae378_Supplemental_File

## Data Availability

The Companion web server is freely available to all users at https://companion.ac.uk. The source code for the Companion pipeline can be found at https://github.com/sii-companion/companion, or from DOI 10.5281/zenodo.11059828. Docker images containing versioned releases of Companion for local containerised running are hosted at https://hub.docker.com/repository/docker/uofgiii/companion.
