# Supplementary material for: Annotation and visualization of parasite, fungi and arthropod genomes with Companion
Source: Nucleic Acids Res. 2024 May 16;52(W1):W39–44. doi: 10.1093/nar/gkae378 (PMC11223846; doi:10.1093/nar/gkae378)
Supplement: gkae378_Supplemental_File [file gkae378_supplemental_file.pdf]

Supplementary Data

Annotation and visualisation of parasite, fungi and arthropod genomes with Companion

William Haese-Hill[1], Kathryn Crouch[1] and Thomas D. Otto[1,2]

[1] School of Infection & Immunity, University of Glasgow, UK
[2] LPHI, CNRS, INSERM, Université de Montpellier, France

Table of Contents

1. Glossary of tools 1
2. Companion workflow 2
3. Web server 3
4. Results 7
   a. Parasite: *Plasmodium* 7
   b. Fungi: *Candida* 10
   c. Vector: *Anopheles* 11
   References 13

1. Glossary of tools

The following is an alphabetical list of all major tools that constitute the Companion pipeline, with a brief description of their function. Their position in the pipeline workflow can be seen in Figure 1.

ABACAS2 (Assefa, et al., 2009) – ordering and orientating nucleotide sequences along a reference

ARAGORN (Laslett and Canback, 2004) – detection of tRNA in nucleotide sequences

AUGUSTUS (Stanke, et al., 2008) – *ab initio* gene prediction with optional extrinsic hints

BRAKER2 (Brůna, et al., 2021) – gene prediction pipeline, incorporating AUGUSTUS and, another gene prediction tool GeneMark-EP+, trained with protein homology evidence

Circos (Krzywinski, et al., 2009) – visualisation tool used for chromosome-level synteny plots. Requires nucmer to generate the synteny. Used in Companion to indicate how similar the assembly is to the reference and how complete the assembly is.

DIAMOND (Buchfink, et al., 2021) – protein sequence aligner, replaced BLASTP due to decrease in runtime.

FastTree (Price, et al., 2009) – phylogenetic tree inference from alignments. Generates Newick file, which is rendered using Phylocanvas. Used to generate the phylogenetic tree visualisation in Companion.

HMMER (Eddy, 2011) – detection of sequence homologs using probabilistic hidden Markov models (HMMs).

INFERNAL (Nawrocki and Eddy, 2013) – inference of RNA features in nucleotide sequence, used for predicting ncRNA.

Liftoff (Shumate and Salzberg, 2021) – lifting over of gene features from reference to target sequence. Faster and more memory-efficient alternative to RATT.

MAFFT (Katoh, et al., 2002) – multiple sequence aligner, used in conjunction with FastTree

Nucmer (Marçais, et al., 2018) – nucleotide sequence alignment program. Part of MUMmer. Used for Circos and ABACAS2.

OrthoFinder (Emms and Kelly, 2019) – detection of gene orthogroups using protein sequences of multiple species. Used to visualise homologous genes between the reference and the annotated sequence.

Pfam (Mistry, et al., 2020) – database of protein families used by HMMER

RATT (Otto, et al., 2011) – transfer of gene models with high synteny from reference to target sequence

2. Companion workflow

In this update paper of Companion we aimed to cater for larger genomics (up to 1 GB), and since several new software tools have been released since the original paper was published (Steinbiss, et al., 2016), we updated some components of the pipeline.

In addition to the original AUGUSTUS and RATT, users are now offered BRAKER2 and Liftoff as default alternatives, respectively (Figure 1).

BRAKER2 is an enhancement of AUGUSTUS with additional support for gene prediction, with consistently improved outcomes at the expense of longer run times. Companion's

reference dataset must now include annotated proteins which are used as input for BRAKER2; these are gathered and formatted automatically by the reference update pipeline (see Section 3 below). BRAKER2 then trains GeneMark-EP+ (Brůna, et al., 2020) and uses AUGUSTUS models pre-trained for each reference species to predict genes. Additional scripts have been incorporated into the Nextflow pipeline to ensure any failure during the running / output-parsing of BRAKER2 is accounted for; base AUGUSTUS is used as a backup in these circumstances. This provides additional stability for what is an inherently complex and multi-faceted addition to the Companion workflow.

Liftoff is a recently released faster and more scalable alternative to RATT which “lifts over” reference annotations from a reference sequence aligned to a target sequence using Minimap2 (Li, 2018). The need for a RATT alternative transpired due to memory scaling issues when running Companion with some larger Vector assemblies (> 500 MB). However, there is still an advantage in using RATT for certain genomes of greater phylogenetic distance to their references, where we still see improved accuracy. There are additional post-processing scripts for Liftoff and RATT to transfer reference rRNA features as a complement to the pre-existing INFERNAL prediction.

Functional annotation has also seen substantial changes. OrthoFinder is a recent alternative to OrthoMCL (Li, et al., 2003) for determining orthologues between two sets of proteins. Where OrthoMCL used BLASTP for protein alignment, OrthoFinder uses DIAMOND which matches BLASTP for sensitivity but is substantially faster. Indeed, the OrthoFinder algorithm consistently achieves higher accuracy in orthologue detection than comparable tools, whether or not the default DIAMOND is used for sequence similarity searches (Emms and Kelly, 2019). OrthoFinder also bypasses the OrthoMCL requirement to store results in an SQL database, simplifying the process.

Additional upgrades leading to faster run times include MUMmer4/nucmer instead of BLASTN for nucleotide alignment when generating Circos plots.

3. Web server

Although a Docker container is available for local operation and allows users to compile their own bespoke reference genome dataset, most users continue to interact with Companion via a web interface. Approximately 300 pulls have been reported by DockerHub in the two years since the repository was created, while there has been an order of magnitude greater number of jobs submitted via the web interface in the same time span (>1,000 in the second half of 2023). This has motivated the continued development of the web server infrastructure and code base to ensure a better user experience.

Source	2016	2019	2023
**amoebadb.org**	*	7	10
**cryptodb.org**	*	10	14
**fungidb.org**	0	112	224
**hostdb.org**	0	0	9
**microsporidiadb.org**	*	23	39
**piroplasmadb.org**	*	10	13
**plasmodb.org**	*	23	25
**toxodb.org**	*	14	15
**tritrypdb.org**	*	30	37
**vectorbase.org**	0	0	52
TOTAL	***62***	***229***	***438***

Table S1 Number of reference organisms available on Companion web server by VEuPathDB site source
** Breakdown unknown; only TOTAL value was quoted in Steinbiss, et al. (2016)*

Companion currently has 438 available references, a 7-fold increase versus the original web server implementation of version 1 (see Table S1). An automated reference update pipeline has been developed (also using Nextflow DSL1) to accommodate this abundance, automatically extracting, formatting, and training models for a given reference domain. The source code is available at https://github.com/sii-companion/reference-update.

Running the script *bin/run_all.sh* on a dedicated data server, reference species files from every VEuPathDB.org (Amos, et al., 2021) domain are automatically downloaded using a Python web service interface (see https://github.com/sii-companion/eupathws), and all files necessary for Companion packaged into a directory. Annotated proteins are gathered from every reference organism of a given site (Table S1) and combined with the relevant OrthoDB v11 clade (Kuznetsov, et al., 2022), to provide a comprehensive pool of protein evidence as input for BRAKER2 (see Section 2 above). The entire reference dataset is then secure copied to the various Companion servers and loaded into each database using a Rake task, which parses unique gene IDs and build numbers to ensure only newly updated/released data is extracted (and so avoid duplication of work). This process occurs biannually.

All references available for selection by the user, together with metadata, is displayed in tabular form at https://companion.gla.ac.uk/references/. Individual reference metadata is also made available as part of the output result files for any completed job.

While the core architecture of the Companion web interface retains the same Ruby on Rails framework as in the first implementation (for version 1), there have been significant developments to improve job concurrency. A MySQL server replaces the previous SQLite database to prevent file locking errors with simultaneous write commands, thus removing

the primary barrier to job concurrency on a single server. The effect of this has enabled the use of a single server for the Companion web interface, housing all available references, where before several servers were deployed to handle groupings of similar references (Protozoa, Fungi, etc). 3 jobs can be run concurrently with ease. To allow efficient processing, our production server has 32 CPU cores, 64 GB RAM, as well as over 1 TB of storage.

Taken together, the core Companion and web server infrastructure changes ensure job completion rates of >95%.

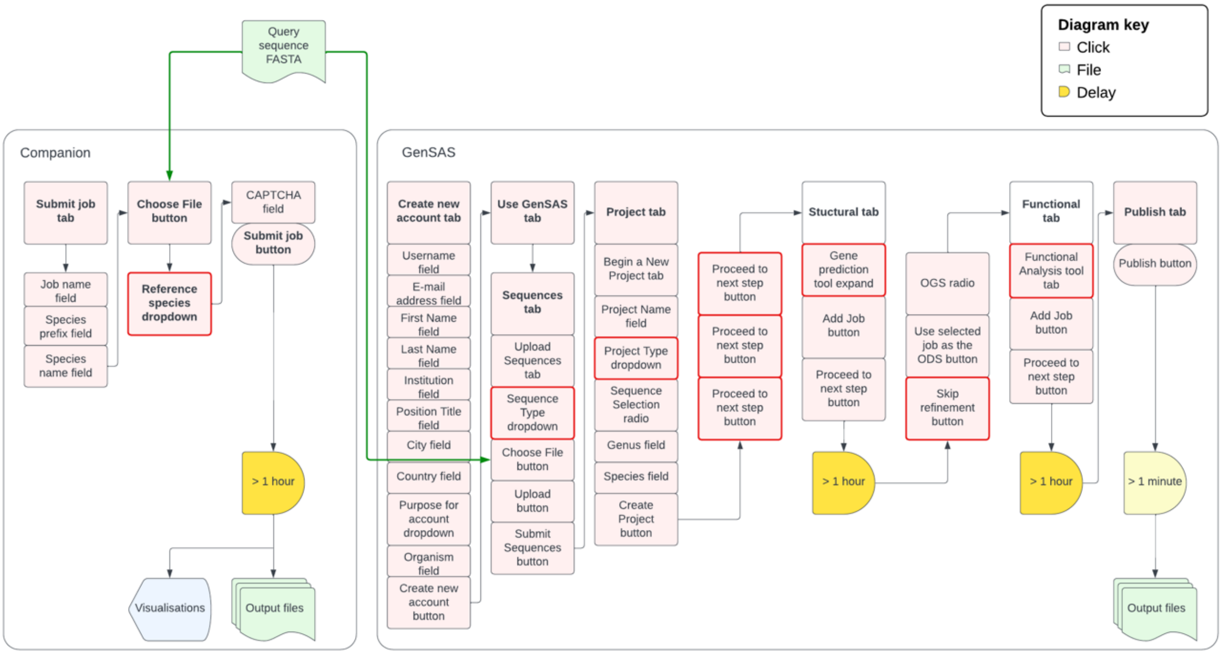

Figure S1 Minimum clicks required for a new user of Companion and GenSAS *to perform a basic job with default settings that ensures both structural and functional annotations: 8 and 41, respectively. Clicks highlighted in red require expert insight about the target genome.*

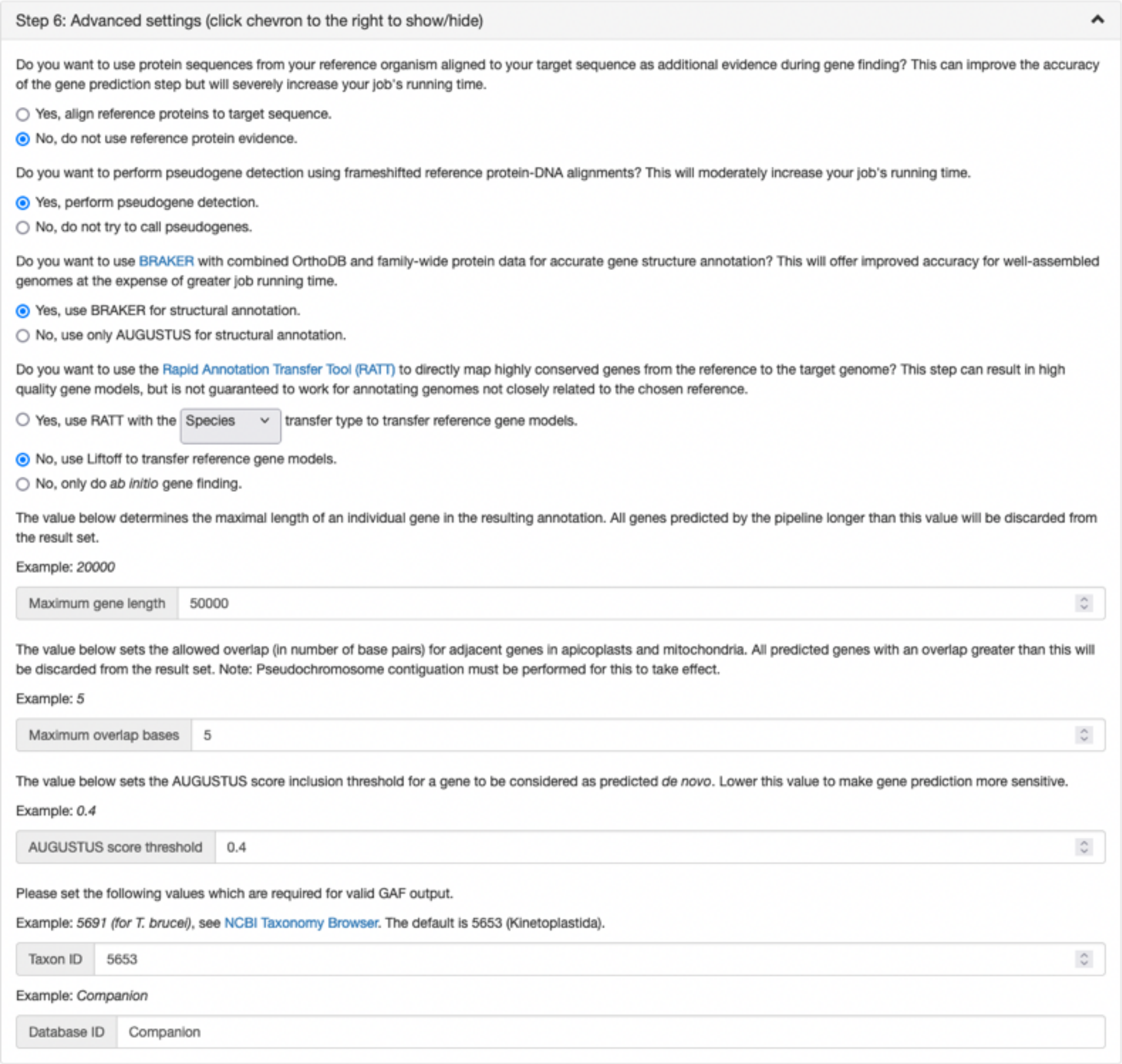

Step 6: Advanced settings (click chevron to the right to show/hide)

Do you want to use protein sequences from your reference organism aligned to your target sequence as additional evidence during gene finding? This can improve the accuracy of the gene prediction step but will severely increase your job's running time.

- ○ Yes, align reference proteins to target sequence.
- ◉ No, do not use reference protein evidence.

Do you want to perform pseudogene detection using frameshifted reference protein-DNA alignments? This will moderately increase your job's running time.

- ◉ Yes, perform pseudogene detection.
- ○ No, do not try to call pseudogenes.

Do you want to use BRAKER with combined OrthoDB and family-wide protein data for accurate gene structure annotation? This will offer improved accuracy for well-assembled genomes at the expense of greater job running time.

- ◉ Yes, use BRAKER for structural annotation.
- ○ No, use only AUGUSTUS for structural annotation.

Do you want to use the Rapid Annotation Transfer Tool (RATT) to directly map highly conserved genes from the reference to the target genome? This step can result in high quality gene models, but is not guaranteed to work for annotating genomes not closely related to the chosen reference.

- ○ Yes, use RATT with the [Species] transfer type to transfer reference gene models.
- ◉ No, use Liftoff to transfer reference gene models.
- ○ No, only do *ab initio* gene finding.

The value below determines the maximal length of an individual gene in the resulting annotation. All genes predicted by the pipeline longer than this value will be discarded from the result set.

Example: *20000*

Maximum gene length: 50000

The value below sets the allowed overlap (in number of base pairs) for adjacent genes in apicoplasts and mitochondria. All predicted genes with an overlap greater than this will be discarded from the result set. Note: Pseudochromosome contiguation must be performed for this to take effect.

Example: *5*

Maximum overlap bases: 5

The value below sets the AUGUSTUS score inclusion threshold for a gene to be considered as predicted *de novo*. Lower this value to make gene prediction more sensitive.

Example: *0.4*

AUGUSTUS score threshold: 0.4

Please set the following values which are required for valid GAF output.

Example: *5691 (for T. brucei)*, see NCBI Taxonomy Browser. The default is 5653 (Kinetoplastida).

Taxon ID: 5653

Example: *Companion*

Database ID: Companion

Figure S2 Companion web server "advanced settings" for optional refinement, *displayed as a collapsable form in the job submission interface.*

4. Results

Target	Source	Reference	Source
***Plasmodium falciparum* Dd2 (22 Mb)**	PlasmoDB release 65	*Plasmodium falciparum* 3D7 (23 Mb)	PlasmoDB release 9
***Candida dubliniensis* CD36 (15 Mb)**	FungiDB release 60	*Candida albicans* SC5314 (14 Mb)	FungiDB release 60
***Anopheles darlingi* assembly idAnoDarlMG-H_01 (177 Mb)**	VectorBase release 65	*Anopheles darlingi* Coari (133 Mb)	VectorBase release 49

Table S2 Data sources for target and Companion reference genomes for three annotation tests. *The approximate size as megabases of each target and reference genome is given in brackets.*

a. Parasite: *Plasmodium*

Companion completed in ~2.5 hours. Selecting options from most of the pre-structural tabs (like repeat masking with EvidenceModeller and alignment with BLASTn), GenSAS took over 6 hours. Excluding these settings, GenSAS completed in ~1 hour. Companion's higher runtime was mostly accounted for by ncRNA prediction with INFERNAL; an intrinsic component of the pipeline. Including tools for ncRNA prediction (RNAmmer and tRNAscan-SE) in the latter GenSAS job, as well as other tools offered by Companion inherently (such as Pfam), equalises the runtime to that of Companion.

To be able to make the *Plasmodium falciparum* Dd2 comparison, we excluded UTR features that were transferred by Liftoff from the reference, as the current annotation did not have them annotated.

Overall, Companion is very effective in annotating the *Plasmodium* genome, despite some over-prediction of genes. Upon investigation, these appear to mostly be in the teleomeric regions where repeats resemble genes. Although these false predictions are easy to detect in tools such as Artemis (Carver, et al., 2012), it would still require manual intervention to obtain a perfect annotation.

Companion was run with default settings (BRAKER2 and Liftoff) and in a second run with RATT (Strain setting), owing to similarity of target and reference (see **Table S**).

Organism	Metric	Companion (Liftoff)	Companion (RATT)
Plasmodium falciparum (5,461 total genes)	Nucleotide accuracy (%)	99.35	99.4
	Exon accuracy (%)	95.05	94.9
	Matching loci (%)	96.22	96.02
	Total genes predicted	5,800	5,791
Candida albicans (6,095 total genes)	Nucleotide accuracy (%)	98.35	98.65
	Exon accuracy (%)	89.50	90.15
	Matching loci (%)	95.54	96.03
	Total genes predicted	6,206	5,833

Table S3 Comparison of standard metrics between two Companion jobs using different gene model transfer tools. *Companion achieves comparably good results when using RATT for gene finding rather than default Liftoff.*

Comparing Companion versions 1 and 2

Performing the same job on legacy Companion (version 1.0.2) (although naturally with AUGUSTUS/RATT rather than BRAKER2/Liftoff), we observed similarly high accuracy to the current annotation, but with a four-fold increase in runtime (~9 hours). As the number of processors was increased from 8 to 32 for the new web server, we observe a near linear decrease in runtime due to parallelisation. Comparisons to the current annotation showed similarly high sensitivity between both runs, but Companion version 2 matched 35 more loci (0.6%).

There have been notable improvements in capturing apicoplast and mitochondrial ncRNA in the latest Companion compared to version 1. The version 2 job captured 4 apicoplast rRNA and 21 mitochondrial rRNA, all of which were omitted in the legacy run of Companion as well as GenSAS (using additional RNAmmer tool in pipeline), see Figure S5.

Overall, the new version of Companion not only outperforms existing tools not built for parasite genomics, but also shows improvement in speed and accuracy to its previous version.

Including RNA-Seq evidence

To assess the impact of additional RNA-Seq evidence we decided to annotate *Plasmodium falciparum Dd2* once more with GenSAS and Companion. Reads were obtained in FASTQ format for run ERR9660878 from the BioStudies database (http://www.ebi.ac.uk/biostudies) under accession number E-MTAB-11679. These were mapped to the *Dd2* reference genome using HISAT2, default parameters, (Kim, et al., 2019) to generate the BAM file input for BRAKER in GenSAS. Cufflinks v2.2.1 (Trapnell, et al., 2012) , default parameters, was run on this BAM file to generate transcript hints in GTF format for use with AUGUSTUS in the Companion pipeline (all other Companion settings were default). The results of these runs can be seen in Table S4. Comparing with the results in

Table 1, there are improvements across both tools, notably a substantial increase in exon accuracy, matching loci, and total genes predicted for GenSAS. However, it is important to note that better results are still observed in Companion, regardless of whether or not additional RNA-Seq evidence is included.

Organism	Metric	GenSAS (BRAKER)	Companion (AUGUSTUS)
Plasmodium falciparum (5,461 total genes)	Nucleotide accuracy (%)	98.40	99.45
	Exon accuracy (%)	87.10	95.95
	Matching loci (%)	85.24	96.31
	Total genes predicted	5,683	5,708

Table S4 Comparison of standard metrics between GenSAS and Companion when using additional RNA-Seq evidence.

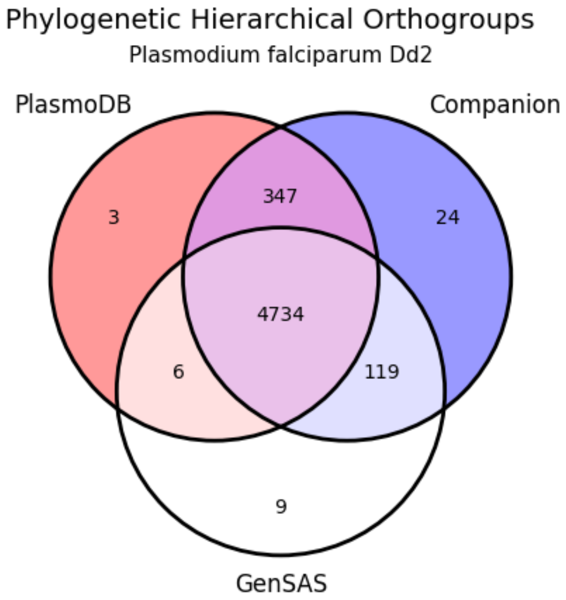

Figure S3 Venn diagram of shared Hierarchical Orthogroups found by OrthoFinder. *Note greater number of orthogroups shared by Companion and current annotation (PlasmoDB): 347, versus only 6 shared exclusively between GenSAS and PlasmoDB. Note also 119 orthogroups suggesting novel genes.*

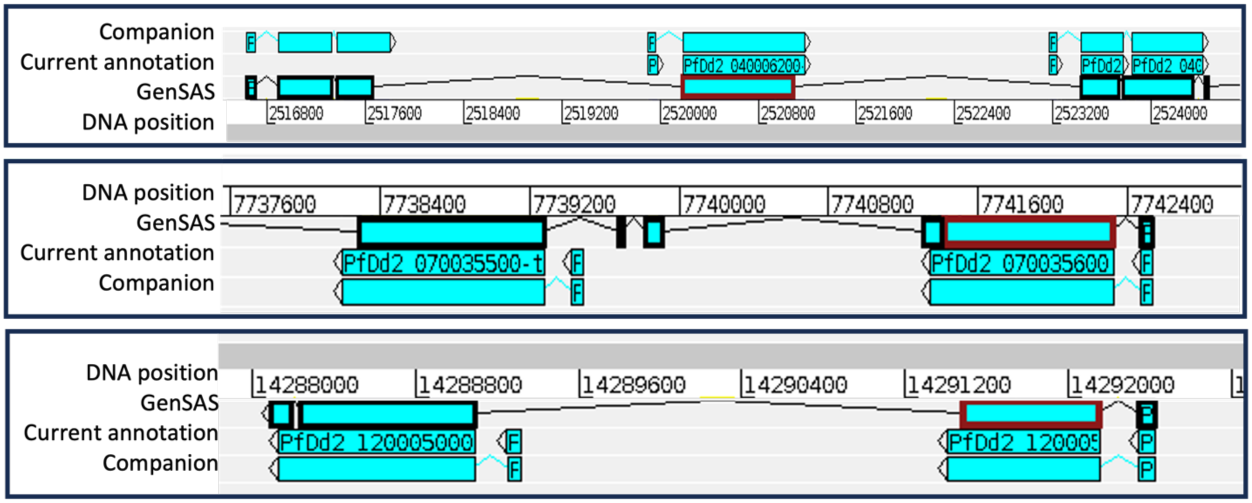

Figure S4 Examples of predicted PfDd2 gene models displayed in Artemis. *GenSAS (inner track) frequently incorrectly merges genes as one (see highlighted CDS with intron bridging).* It also misses smaller exons. Interestingly, in the first panel, the current annotation has a missing gene.

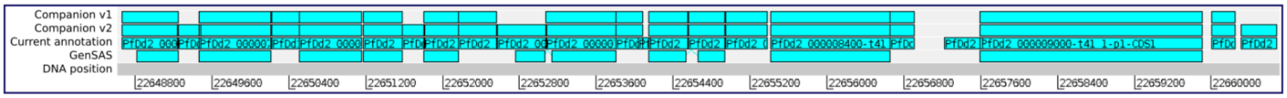

Figure S5 Examples of improved apicoplast gene prediction by Companion version 2. *Several genes matched by Companion version 2 (v2) and current annotation (middle two tracks) but missed by GenSAS (bottom track) and Companion version 1 (v1). The two genes missed by Companion v2, PfDd2_000008050 and PfDd2_000008950, were due to an overlap with an adjacent gene of 34 and 16 bases, respectively; outside the default value of Companion's new maximum overlap setting (see Figure S2).*

b. Fungi: *Candida*

Both Companion and GenSAS jobs completed in approximately 1 hour. Like before, additional pipeline tools in GenSAS (such as RepeatModeller, BLASTn alignment) resulted in much greater run times for modestly worse performance overall. Output visualisations from Companion can be seen in Figure S6.

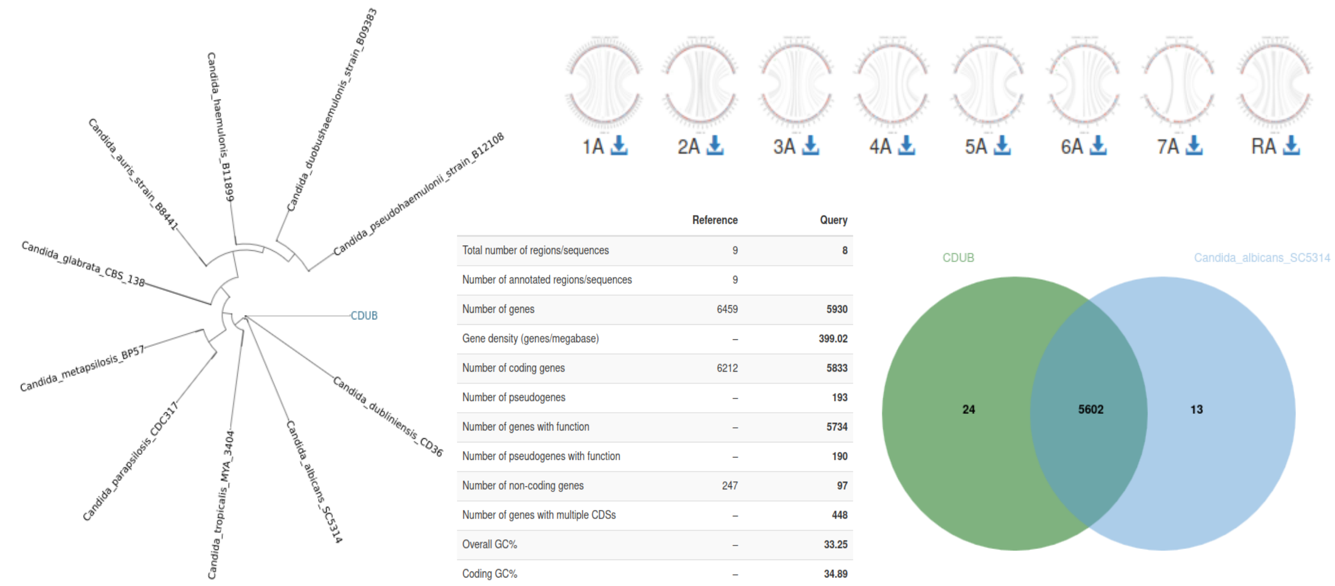

	Reference	Query
Total number of regions/sequences	9	8
Number of annotated regions/sequences	9	
Number of genes	6459	5930
Gene density (genes/megabase)	–	399.02
Number of coding genes	6212	5833
Number of pseudogenes	–	193
Number of genes with function	–	5734
Number of pseudogenes with function	–	190
Number of non-coding genes	247	97
Number of genes with multiple CDSs	–	448
Overall GC%	–	33.25
Coding GC%	–	34.89

Figure S6 Visualisations of fungi example job from Companion web server*, showing radial phylogenetic tree, synteny, orthology and statistics.*

c. Vector: *Anopheles*

Finally, to highlight the expansion of Companion to allow larger vector genomes, we decided to annotate an unpublished *Anopheles darlingi* assembly (idAnoDarlMG-H_01, accession PRJEB53253) against the same-species reference *Anopheles darlingi* Coari (Marinotti, et al., 2013).

Companion was once again run with BRAKER2 for structural annotation. This time, Liftoff was an obvious choice over RATT to account for the better memory scaling of Liftoff for larger genomes (see Section 2 above) and its impressive performance lifting over genes from target and reference of high similarity. The GenSAS AUGUSTUS training set only includes two vector species: *Aedes aegypti* and *Drosophila melanogaster*, so we were forced once more to use GeneMarkES (*ab initio*) for structural annotation. The use of GeneMarkES ensured that the GenSAS job completed in ~3 hours versus the Companion job's ~9 hours runtime. The addition of ncRNA prediction tools to the GenSAS pipeline (RNAmmer and tRNAscan-SE) added an additional ~1 hour to the total runtime.

Mitochondrial gene transfer was also carried out by Companion. All 13 orthologous mRNA were transferred from the reference AdarC3_MT sequence, and an additional 2 tRNA were predicted. None of these non-coding RNA are available in the current annotation, nor were they predicted by GenSAS (despite the addition of ncRNA prediction tools to the pipeline).

Table 1 metrics show generally favourable results for Companion versus GenSAS, although neither tool achieves comparably high outcomes to the previous tests, implying there's still

work to be done in improving Vector annotation more generally. It is important to note that quality annotation of Vector genomes is relatively sparse (symbolically, the study for the chosen assembly remains unpublished), and so the notion of such a reference annotation being a valid "truth set" comparison is questionable. This motivated running GFFCompare (Pertea and Pertea, 2020) after filtering for only CDS features in both the reference and query annotations, where the inclusion of UTRs in the current annotation appeared to significantly hinder the metrics for both tools.

A method of validation that doesn't require reference annotation is BUSCO completeness (Seppey, et al., 2019), which assesses protein sequences against a database of proteins from a relevant lineage. Using lineage database insecta_odb10, proteins output by Companion achieved 95.4% complete (C), 0.7% fragmented (F) and 3.9% missing (M). This compares favourably to the GenSAS output which achieved 94.1% C, 1.1% F and 4.8% M. The more specific lineage database diptera_odb10 saw scores of 93.9% C, 1.4% F and 4.7% M for Companion, versus comparable results of 93.2% C, 1.7% F and 5.1% M for GenSAS. The current annotation idAnoDarlMG-H_01 assembly proteins (from VectorBase) achieved 99.1% C, 0.1% F and 0.8% M against diptera_odb10, for comparison.

Another alternative method of validation that also considers proteins is coverage of genes that contain a Pfam domain annotation (this was used successfully in Holt and Yandell (2011)). The results, including a dissection of some of the more prominent functions, can be observed in Figure S7**Error! Reference source not found.**. There is a modest improvement in Companion of 2.2% against GenSAS. The addition of InterProScan to perform protein matching of Pfam domains added ~10.5 hours to the overall GenSAS runtime. HMMER v3.3 (the same as used in the Companion pipeline) was run independently on the current annotation proteins to determine their Pfam coverage.

It should be noted that we tested various approaches on the command line to improve the annotation, using different input of RNA-Seq data, training with proteins of the Vector domain only. However, Companion always returned the best annotation results.

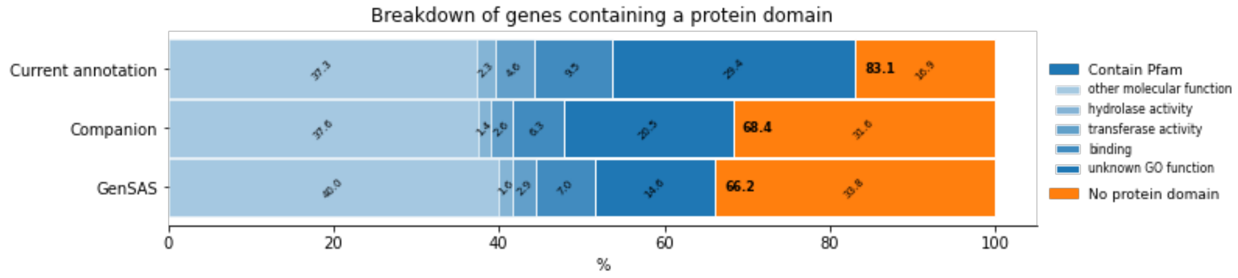

Figure S7 Percentage of genes that were annotated with a Pfam domain.

References

Amos, B., *et al.* VEuPathDB: the eukaryotic pathogen, vector and host bioinformatics resource center. *Nucleic Acids Research* 2021;50(D1):D898-D911.
Assefa, S., *et al.* ABACAS: algorithm-based automatic contiguation of assembled sequences. *Bioinformatics* 2009;25(15):1968-1969.
Brůna, T., *et al.* BRAKER2: automatic eukaryotic genome annotation with GeneMark-EP+ and AUGUSTUS supported by a protein database. *NAR Genomics and Bioinformatics* 2021;3(1).
Brůna, T., Lomsadze, A. and Borodovsky, M. GeneMark-EP+: eukaryotic gene prediction with self-training in the space of genes and proteins. *NAR Genom Bioinform* 2020;2(2):lqaa026.
Buchfink, B., Reuter, K. and Drost, H.-G. Sensitive protein alignments at tree-of-life scale using DIAMOND. *Nature Methods* 2021;18(4):366-368.
Carver, T., *et al.* Artemis: an integrated platform for visualization and analysis of high-throughput sequence-based experimental data. *Bioinformatics* 2012;28(4):464-469.
Eddy, S.R. Accelerated Profile HMM Searches. *PLOS Computational Biology* 2011;7(10):e1002195.
Emms, D.M. and Kelly, S. OrthoFinder: phylogenetic orthology inference for comparative genomics. *Genome Biology* 2019;20(1):238.
Holt, C. and Yandell, M. MAKER2: an annotation pipeline and genome-database management tool for second-generation genome projects. *BMC Bioinformatics* 2011;12(1):491.
Katoh, K., *et al.* MAFFT: a novel method for rapid multiple sequence alignment based on fast Fourier transform. *Nucleic Acids Research* 2002;30(14):3059-3066.
Kim, D., *et al.* Graph-based genome alignment and genotyping with HISAT2 and HISAT-genotype. *Nature Biotechnology* 2019;37(8):907-915.
Krzywinski, M., *et al.* Circos: an information aesthetic for comparative genomics. *Genome Res* 2009;19(9):1639-1645.
Kuznetsov, D., *et al.* OrthoDB v11: annotation of orthologs in the widest sampling of organismal diversity. *Nucleic Acids Research* 2022;51(D1):D445-D451.
Laslett, D. and Canback, B. ARAGORN, a program to detect tRNA genes and tmRNA genes in nucleotide sequences. *Nucleic Acids Research* 2004;32(1):11-16.
Li, H. Minimap2: pairwise alignment for nucleotide sequences. *Bioinformatics* 2018;34(18):3094-3100.
Li, L., Stoeckert, C.J., Jr. and Roos, D.S. OrthoMCL: identification of ortholog groups for eukaryotic genomes. *Genome Res* 2003;13(9):2178-2189.
Marçais, G., *et al.* MUMmer4: A fast and versatile genome alignment system. *PLoS Comput Biol* 2018;14(1):e1005944.
Marinotti, O., *et al.* The genome of Anopheles darlingi, the main neotropical malaria vector. *Nucleic Acids Res* 2013;41(15):7387-7400.
Mistry, J., *et al.* Pfam: The protein families database in 2021. *Nucleic Acids Research* 2020;49(D1):D412-D419.
Nawrocki, E.P. and Eddy, S.R. Infernal 1.1: 100-fold faster RNA homology searches. *Bioinformatics* 2013;29(22):2933-2935.
Otto, T.D., *et al.* RATT: Rapid Annotation Transfer Tool. *Nucleic Acids Res* 2011;39(9):e57.
Pertea, G. and Pertea, M. GFF Utilities: GffRead and GffCompare [version 2; peer review: 3 approved]. *F1000Research* 2020;9(304).
Price, M.N., Dehal, P.S. and Arkin, A.P. FastTree: Computing Large Minimum Evolution Trees with Profiles instead of a Distance Matrix. *Molecular Biology and Evolution* 2009;26(7):1641-1650.
Seppey, M., Manni, M. and Zdobnov, E.M. BUSCO: Assessing Genome Assembly and Annotation Completeness. *Methods Mol Biol* 2019;1962:227-245.
Shumate, A. and Salzberg, S.L. Liftoff: accurate mapping of gene annotations. *Bioinformatics* 2021;37(12):1639-1643.
Stanke, M., *et al.* Using native and syntenically mapped cDNA alignments to improve de novo gene finding. *Bioinformatics* 2008;24(5):637-644.
Steinbiss, S., *et al.* Companion: a web server for annotation and analysis of parasite genomes. *Nucleic Acids Research* 2016;44(W1):W29-W34.
Trapnell, C., *et al.* Differential gene and transcript expression analysis of RNA-seq experiments with TopHat and Cufflinks. *Nat Protoc* 2012;7(3):562-578.
